# Thrombosis and Vascular Inflammation in Diabetes: Mechanisms and Potential Therapeutic Targets

**DOI:** 10.3389/fcvm.2018.00001

**Published:** 2018-01-19

**Authors:** Nikoletta Pechlivani, Ramzi A. Ajjan

**Affiliations:** ^1^School of Medicine, Leeds Institute for Cardiovascular and Metabolic Medicine, University of Leeds, Leeds, United Kingdom

**Keywords:** atherosclerosis, diabetes mellitus, thrombosis, endothelial dysfunction, vascular inflammation

## Abstract

Cardiovascular disease remains the main cause of morbidity and mortality in patients with diabetes. The risk of vascular ischemia is increased in this population and outcome following an event is inferior compared to individuals with normal glucose metabolism. The reasons for the adverse vascular profile in diabetes are related to a combination of more extensive atherosclerotic disease coupled with an enhanced thrombotic environment. Long-term measures to halt the accelerated atherosclerotic process in diabetes have only partially addressed vascular pathology, while long-term antithrombotic management remains largely similar to individuals without diabetes. We address in this review the pathophysiological mechanisms responsible for atherosclerosis with special emphasis on diabetes-related pathways. We also cover the enhanced thrombotic milieu, characterized by increased platelet activation, raised activity of procoagulant proteins together with compromised function of the fibrinolytic system. Potential new therapeutic targets to reduce the risk of atherothrombosis in diabetes are explored, including alternative use of existing therapies. Special emphasis is placed on diabetes-specific therapeutic targets that have the potential to reduce vascular risk while keeping an acceptable clinical side effect profile. It is now generally acknowledged that diabetes is not a single clinical entity but a continuum of various stages of the condition with each having a different vascular risk. Therefore, we propose that future therapies aiming to reduce vascular risk in diabetes require a stratified approach with each group having a “stage-specific” vascular management strategy. This “individualized care” in diabetes may prove to be essential to improve vascular outcome in this high risk population.

## Introduction

The prevalence of diabetes continues to increase and is reaching epidemic proportions.^1^ The vast majority of these patients have type 2 diabetes characterized early in the disease process by insulin resistance (IR) followed by pancreatic β-cell failure leading to elevated blood glucose and emergence of diabetes (1–3). A minority have type 1 diabetes, secondary to immune destruction of the β-cells. However, these individuals can also develop IR leading to double diabetes, which has features of both type 1 and type 2 diabetes (4), and this group appears to have an enhanced vascular risk.

Despite advances in therapy, cardiovascular disease (CVD) remains the main cause of morbidity and mortality in diabetes (5, 6). Similarly to CVD in individuals without diabetes, an increased inflammatory response and an enhanced thrombotic milieu represent the two main pathological pathways underpinning atherothrombosis in diabetes. However, the adverse vascular process is accelerated in diabetes, secondary to a combination of IR and elevated blood glucose levels. Understanding the exact mechanisms involved in disease initiation and progression is crucial in order to develop effective vascular preventative and therapeutic treatment strategies (7–9).

This review summarizes the pathophysiological mechanisms responsible for increased atherothrombotic risk in diabetes, addresses current treatment modalities, and explores additional pathways that can be targeted to reduce vascular events in this high-risk population.

### Pathophysiology

Both extensive vascular pathology and an enhanced thrombotic environment contribute to premature vascular occlusive events and poor clinical outcome in patients with diabetes.

A normal endothelial cell (EC) function is the key to maintain vascular health. EC dysfunction is one of the earliest abnormalities in the atherothrombotic process, which also contributes to the latter stages of the disease. Another central mechanism in vascular pathology is systemic inflammation, which promotes vascular damage. EC dysfunction and an inflammatory milieu are both associated with a prothrombotic and hypofibrinolytic environment, facilitating vascular occlusion and leading to myocardial infarction, stroke, and occlusive peripheral vascular disease, common complications in patients with diabetes.

#### EC Dysfunction

The endothelium plays an important role in maintaining vascular homeostasis by regulating vasodilation and vasoconstriction, thrombosis and fibrinolysis, platelet activation, platelet and leukocyte interaction, and smooth muscle cell function. Under physiological conditions, the endothelium modulates vascular tone by producing and releasing various vasodilator substances, most importantly nitric oxide (NO), and vasoconstrictor substances including endothelin (10, 11). When EC dysfunction occurs, secondary to IR with or without elevated blood glucose levels, vascular homeostasis is disturbed and the process of atherosclerosis ensues (11, 12). EC dysfunction results in these cells expressing adhesion molecules, thus attracting inflammatory cells. Moreover, EC dysfunction disrupts the barrier function of these cells causing the movement of low-density lipoprotein cholesterol (LDL) from vessel lumen into the wall, where it is oxidized to the highly atherogenic ox-LDL. This molecule is then taken up by inflammatory cells that moved from the blood stream to the vessel wall secondary to increased permeability of dysfunctional EC. The uptake of ox-LDL by macrophages results in the formation of foam cells, which aggregate together to form the fatty streak, the earliest abnormality in the atherosclerotic process, and which can be found as early as childhood. An inflammatory reaction is followed by deposition of collagen, gradually transforming the healthy vessel wall into a series of atherosclerotic plaques. Once the atherosclerotic plaque ruptures, it exposes a prothrombotic core resulting in activation of platelets and the protein phase of coagulation, culminating in the formation of the obstructive vascular clot.

**Figure 1:**
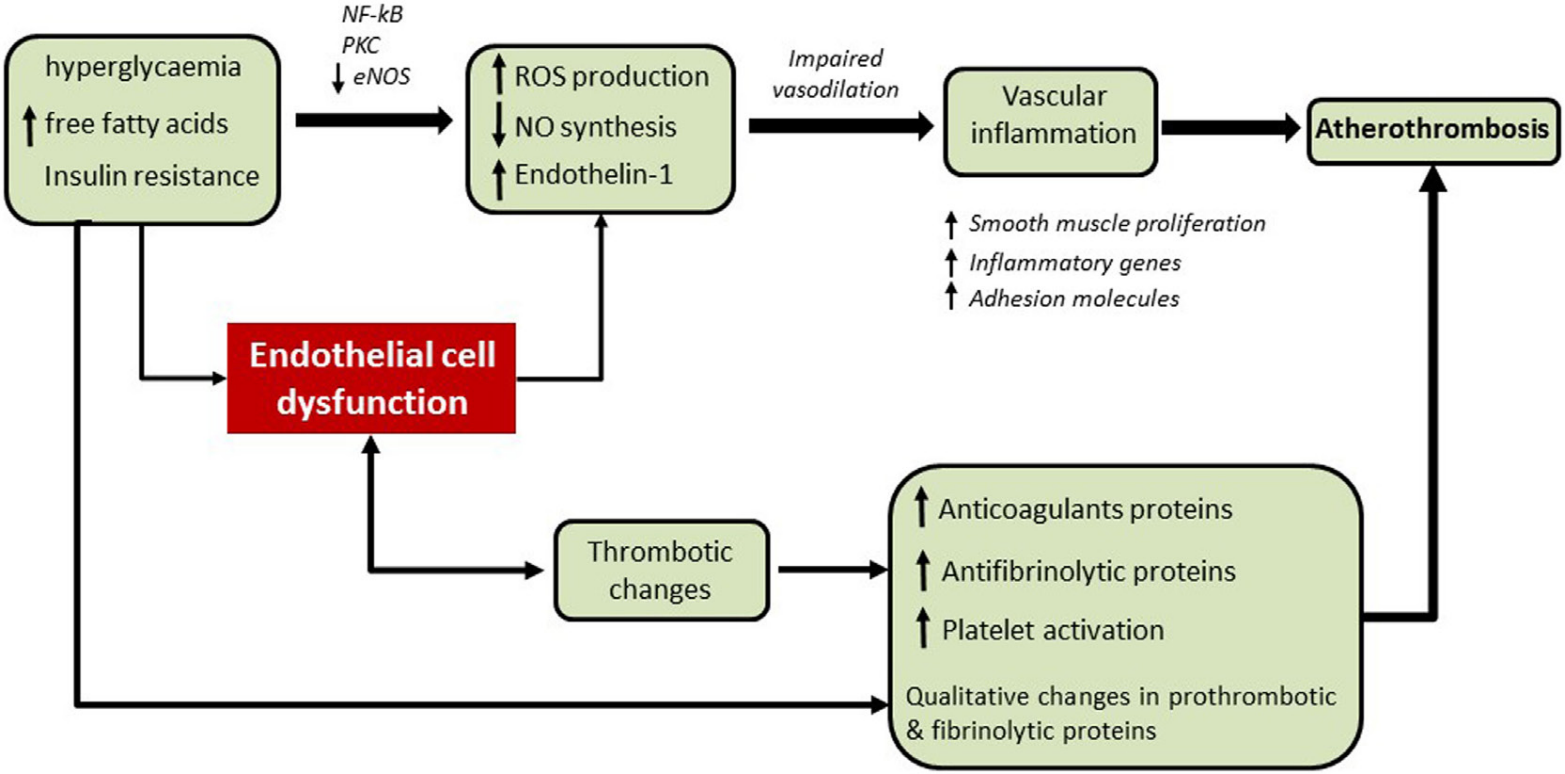
The accelerated vascular pathology and enhanced thrombotic environment in diabetes. Endothelial dysfunction plays a key role in all stages of the atherosclerotic process. In the early stages of the disease, insulin resistance and increased levels of glucose and free fatty acids enhance the production of reactive oxygen species (ROS) and reduce nitric oxide (NO) synthesis *via* several mechanisms including activation of NF-κB and protein kinase C (PKC) signaling and reduction of endothelial NO synthase (eNOS) activity. Endothelial dysfunction contributes to the impairment of vasodilation, expression of adhesion molecules, and further vascular inflammation. In the latter stages of the disease, endothelial dysfunction results in increased platelet activation and a prothrombotic/hypofibrinolytic environment which facilitates vascular occlusion and atherothrombosis.

Endothelial cell dysfunction plays a role in all stages of the atherosclerotic process from initiation of atherosclerosis to precipitation of thrombosis (10, 13), as summarized in Figure 1. Therefore, therapy targeted at improving the health of these cells would help to reduce the risk of atherothrombosis. In diabetes, this includes general measures, such as ensuring good glycemic control, reducing IR, and optimizing blood pressure control. However, there are additional steps that can be undertaken to target specific pathways, which are further detailed below.

#### Vascular Inflammation and Atherosclerosis in Diabetes

We will concentrate on diabetes-specific pathways for vascular pathology related to IR and elevated glucose levels.

Reduced NO bioavailability and elevated levels of reactive oxygen species (ROS) play fundamental roles in vascular disease in diabetes (Figure 1). IR inhibits NO production by decreasing the activity of endothelial NO synthase (eNOS) resulting in reduced vasodilation (14–16). In addition to reduced production of vasodilators, there is an increased production of vasoconstrictors in diabetes. For example, the vasoconstrictor endothelin-1 is involved with endothelial dysfunction and increased plasma levels have been associated with microangiopathy in type 2 diabetes (17). Furthermore, increased arterial stiffness in diabetes has been associated with phenotype switching of vascular smooth muscle cells, a process that appears to be controlled by microRNAs (miRNAs); *mi*R-145 has recently been shown to modulate the phenotypic switch of vascular smooth muscle cells from a contractile to a proliferative state in atherosclerosis (18, 19).

Decreased NO bioavailability has also been related to platelet activation. An *in vivo* study in diabetic mice has demonstrated that inhibiting NO synthase reduced platelet vasodilator-stimulated phosphoprotein (VASP) phosphorylation and increased fibrinogen-platelet binding and expression of P-selectin as well as CD40 ligand. Diabetic mice also exhibited reduced VASP phosphorylation, increased fibrinogen-platelet binding, and enhanced expression of P-selectin/CD40 ligand, which was rescued by endothelial-specific restoration of NO production (20). This emphasizes the importance of NO production by ECs in controlling platelet activation, a process that is compromised in the presence of endothelial dysfunction.

Hyperglycemia in diabetes and elevated levels of free fatty acids enhance ROS production, which in turn compromises NO synthesis *via* a number of cellular mechanisms. More specifically, free fatty acids bind to Toll-like receptor, activating NF-κB, which stimulates inflammation by increasing the expression of the inflammatory molecules interleukin (IL)-6 and tumor necrosis factor (TNF)-α. Moreover, the stimulation of the toll-like receptor induces the phosphorylation of insulin receptor substrate-1 by c-Jun amino-terminal kinase (JNK) and protein kinase C (PKC) causing downregulation of the PI3-kinase/Akt pathway and the glucose transporter GLUT-4. Suppression of the PI3-kinase/Akt pathway leads to reduced eNOS activity and decreased NO production. Furthermore, the increased oxidative stress and hyperglycemia, stimulate vascular inflammation *via* several cellular mechanisms, including promoting activation of PKC and NF-κB signaling. Secretion of cytokines IL-1 and TNF-α enhances NF-κB activity and production of adhesion molecules by ECs further aggravating the inflammatory process (21, 22).

**Figure 2:**
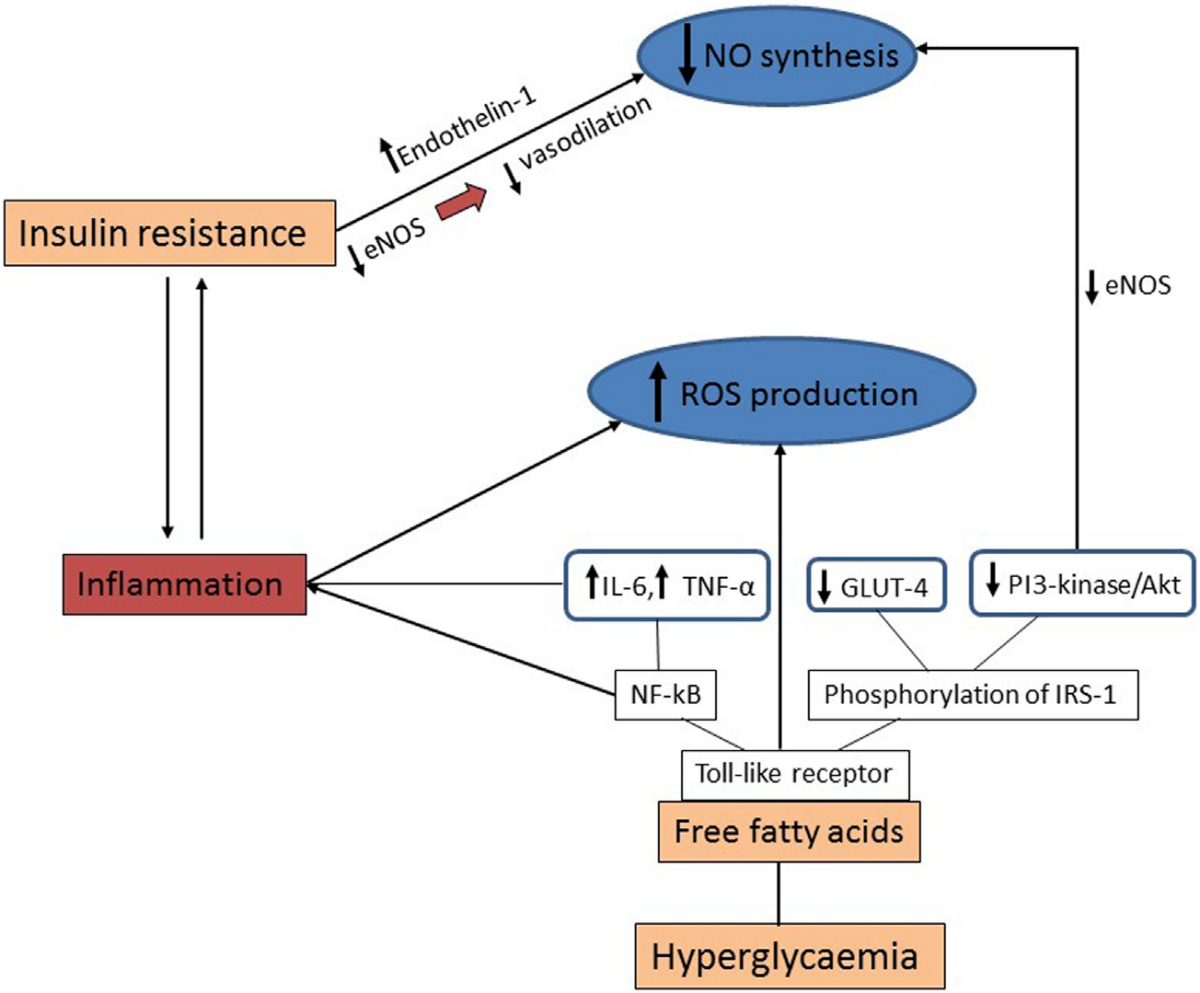
Mechanistic pathways for increased vascular inflammation in diabetes. Insulin resistance inhibits nitric oxide (NO) synthesis by reducing vasodilation *via* decreased activity of endothelial NO synthase (eNOS) together with increased production of vasoconstrictors such as endothelin-1. Hyperglycemia and increased levels of free fatty acids enhance the production of reactive oxygen species (ROS) and reduce NO synthesis *via* several cellular mechanisms. Free fatty acids bind to toll-like receptor which activates the NF-κB pathway and stimulates inflammation by increasing interleukin (IL)-6 and tumor necrosis factor (TNF)-α. Additionally, toll-like receptor stimulates the phosphorylation of insulin receptor substrate-1 (IRS-1) followed by downregulation of the PI3-kinase/Akt pathway and the glucose transporter GLUT-4, consequently resulting in reduction of NO production.

Figure 2 summarizes the main mechanistic pathways operating to increase vascular inflammation in diabetes.

#### Thrombotic Changes in Diabetes

The metabolic changes including IR and hyperglycemia are associated with both increased platelet activation and reduced response to antiplatelet therapy, which have been extensively reviewed elsewhere (23–26). Briefly, there are two main types of antiplatelet agents that are currently used for long-term therapy in diabetes. The first targets the thromboxane pathway, represented by aspirin, whereas the second targets the P2Y12 pathway, and this family of drugs currently includes three agents: clopidogrel, prasugrel, and ticagrelor. More antiplatelet agents are in development that target additional pathways (26).

Following myocardial infarction, dual antiplatelet therapy (DAPT) is used employing agents that target the thromboxane and P2Y12 pathways. However, inhibitors of the P2Y12 pathway seem to vary in efficacy. In the TRITON trial, DAPT with aspirin and prasugrel following myocardial infarction was superior to aspirin and clopidogrel but at the expense of increased bleeding events, failing to overall improve clinical outcome. A sub-analysis of diabetes patients, however, has shown reduction in further ischemic events without a significant increase in bleeding risk (27), making prasugrel particularly useful for patients with diabetes. The newer P2Y12 inhibitor ticagrelorhas also shown both biochemical and clinical superiority to clopidogrel when used in combination with aspirin following myocardial infarction, an effect that was observed in both diabetes and non-diabetes patients without an increase in bleeding risk (28–31). Moreover, the PEGASUS-TIMI 54 trial showed that ticagrelor addition to background aspirin therapy in patients with stable coronary artery disease caused significant reduction of cardiovascular risk compared with the placebo group (32). The previously documented reduced efficacy of clopidogrel in diabetes does not seem to apply to the newer agent ticagrelor, showing the progress made at inhibiting P2Y12 pathway in diabetes. In contrast, the inhibition of the thromboxane pathway by aspirin remains an area of debate. Given the short half-life of aspirin and the increased platelet turnover in this condition, the use of twice daily aspirin in diabetes has been advocated. Indeed, clinical studies have shown a significant reduction in platelet reactivity to thromboxane when using aspirin twice daily vs once/day (33–36). However, it remains unclear whether twice daily aspirin affects clinical outcome and studies in this area are lacking.

A relatively recent development is the implication of reticulated platelets in atherothrombotic events (37). These are immature platelets that show resistance to the action of antiplatelet therapy (38, 39). However, the degree of resistance appears to differ comparing antiplatelet agents, with ticagrelor the least affected (40). Interestingly, a recent study has shown that poorly controlled diabetes is characterized by increased circulating reticulated platelets through induction of an inflammatory pathway that leads to increased thrombopoietin production. A correlation between HbA1c and reticulated platelets was found in patients with T2DM and improving glycemic control, using an agent in the sodium glucose co-transporter-2 (SGLT2) inhibitor family, reduced reticulated thrombocytosis in mice. These findings indicate that one strategy for improved efficacy of antiplatelet agents is to reduce circulating reticulated platelets, which can be achieved by lowering glucose levels using SGLT2 inhibitors. This in turn may help to reduce cardiovascular risk in patients with diabetes (41). Indeed, studies on SGLT2 inhibitors have shown reduction in the composite end point of myocardial infarction, stroke, and cardiovascular death (42) and controlling platelet reactivity may be one of the mechanisms involved.

In addition to enhanced platelet activation, diabetes is associated with increased plasma levels and/or activity of various coagulation factors. The net result is an enhanced susceptibility to forming fibrin networks which are characterized by increased density and resistance to fibrinolysis (43, 44). For example, tissue factor (TF) and factor VII (FVII) levels are increased in diabetes, which in turn explains the enhanced production of thrombin leading to higher risk of clot formation. Moreover, plasma levels of fibrinogen are increased in diabetes, as part of the ongoing low-grade inflammation, which contributes to formation of denser clots. In addition, anticoagulants, such as thrombomodulin and protein C, are reduced in diabetes further predisposing to the prothrombotic environment (43, 45). A key antifibrinolytic protein is plasminogen activator inhibitor-1 (PAI-1), which inhibits conversion of plasminogen to active plasmin. Plasma levels of PAI-1 are increased in diabetes, thereby impairing the fibrinolytic process. It should be noted that patients with type 2 diabetes, but not type 1 diabetes, have increased levels of PAI-1 indicating that IR, rather than hyperglycemia *per se*, drives the increased production of this antifibrinolytic protein, a concept that was confirmed using *in vitro* and *in vivo* studies (9, 44, 46, 47). Therefore, ameliorating IR would have more of an effect on PAI-1 levels than simply improving glycemia in diabetes, highlighting some of the complexities involved in the management of this condition.

In addition to quantitative changes, qualitative abnormalities have been documented in diabetes that increase thrombosis risk. This includes altered fibrinogen posttranslational modifications, including increased glycation and oxidation, resulting in denser fibrin networks (48–51). Also, we have shown that hyperglycemia can directly affect the fibrinolytic system by increasing plasminogen glycation, which impairs conversion to plasmin and adversely affects protein activity. Interestingly, a relatively modest decrease in plasma glucose levels improves plasminogen function, demonstrating the importance of optimizing glycemic control in these patients (52).

**Figure 3:**
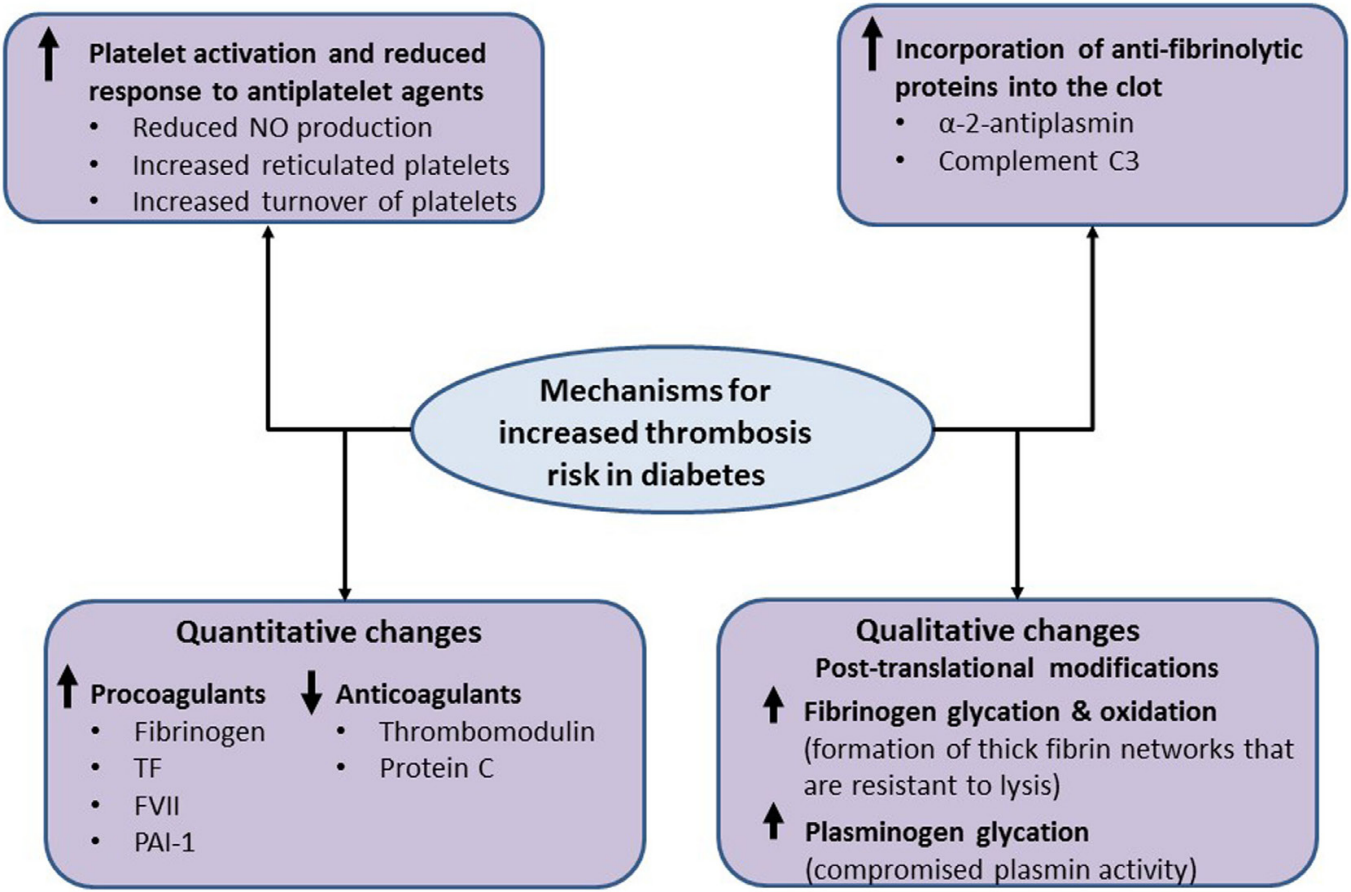
Mechanistic pathways for increased thrombosis risk in diabetes. The metabolic changes in diabetes are associated with increased platelet activation, secondary to reduced nitric oxide (NO) production, an increase in reticulated platelets and increased platelet turnover, which can be only partially controlled by antiplatelet therapy. Diabetes is also associated with increased plasma levels of procoagulants and the antifibrinolytic protein levels, including fibrinogen, tissue factor (TF), factor VII (FVII), and plasminogen activator inhibitor-1 (PAI-1) as well as decreased levels of anticoagulants, including thrombomodulin and protein C. Moreover, qualitative changes in coagulation proteins, including increased oxidation and glycation of fibrinogen, have also been reported in diabetes, which result in the formation of thick fibrin networks that are resistant to lysis. Increased glycation of plasminogen compromises conversion to plasmin and alters protein activity. Finally, increased incorporation of antifibrinolytic proteins, such as α 2-antiplasmin and complement C3, into the clot impairs fibrinolysis and may represent a new therapeutic approach to reduce thrombosis risk in diabetes.

An interesting, and potentially clinically relevant, mechanism for impaired fibrin clot lysis in diabetes is focused on increased incorporation of antifibrinolytic proteins into the clot. Alpha 2-antiplasmin is cross-linked into the fibrin clot and it inhibits plasmin by forming a stable complex with this protein. It has been reported that type 1 diabetes patients have increased alpha 2-antiplasmin incorporation into their fibrin clots (47, 53). Similarly, we have shown that complement C3 incorporation into diabetes clots is increased in diabetes and directly prolongs clot lysis (54, 55). Moreover, C3 plasma levels were independent predictors of clot lysis in a large cohort of type 2 diabetes patients (56, 57). Taken together, modulating incorporation of antifibrinolytic proteins into diabetes clots may represent a new therapeutic strategy to reduce thrombotic risk in diabetes. The main mechanisms operating to increase thrombosis risk and may represent future therapeutic targets in diabetes are summarized in Figure 3.

### From Bench to Bedside

Given the pathophysiological mechanisms involved in vascular pathology in diabetes, there are two main areas to target: slowing the progression of atherosclerosis and reducing the prothrombotic/hypofibrinolytic environment. Therefore, current therapies are directed at improving endothelial function, reducing systemic inflammation, and controlling the enhanced thrombotic environment.

### Targeting Atherosclerosis

In general, improving glycemia and reducing IR, which is part of the clinical management strategy in diabetes, have key roles in halting the accelerated atherosclerotic process in this condition.

A number of existing agents, and others in development, target the inflammatory pathway to reduce atherosclerosis risk. Statins, known for their ability to decrease cholesterol levels, have also been shown to affect the inflammatory response and this dual mode of action explains the efficacy of this class of drugs in reducing vascular events in diabetes (58–60).

The initial enthusiasm with preclinical studies involving the inhibitor of phospholipase A2 (PLA2) darapladip quickly faded with the failure of this agent to reduce coronary vascular events. The clinical findings suggest that this enzyme is a biomarker of vascular pathology rather than a factor having a direct role in pathogenesis. Inadequate dosing and off-target effect(s) have also been blamed for failing to demonstrate a clinical benefit with this agent so far (61). More studies are currently ongoing that will fully clarify the role of PLA2 as an agent that modifies the atherosclerotic process (62, 63).

Experimental studies and preclinical models have indicated that IL-1β blockade is effective at preventing coronary artery disease. A recent study involving 10,061 patients with previous myocardial infarction and C-reactive protein levels above 2 mg/l, showed that IL-1 inhibition, using Canakinumab, reduced the composite end point of non-fatal myocardial infarction, non-fatal stroke, and cardiovascular death by 15 and 14% using 150 and 300 mg of this agent, respectively (injected once every 3 months). However, there was no difference in all-cause mortality, which may be related to increased fatal infection in the Canakinumab arm of the study.

Antioxidants have been repeatedly considered as potential drugs for the prevention and therapy of atherosclerosis given the role of ROS in the pathogenesis of atherosclerosis (64). However, clinical studies with antioxidants have been generally disappointing; a well-conducted double-blind, randomized, placebo-controlled study involving 6,144 patients with established CVD has shown that the antioxidant succinobucol (AGI-1067) has no beneficial effect on vascular outcome, dampening the enthusiasm toward such therapy (65). The generally negative results with various antioxidant therapies suggest that targeting a single pathway involved in vascular pathology is perhaps not enough for translation into clinical benefit. The establishment of the European consortium for the study of ROS should further help to shed more light on the role of this pathway as a therapeutic target in vascular pathology, including high-risk conditions such as diabetes (66).

Given the pivotal role of endothelial dysfunction in vascular pathology, a number of agents that improve EC function are currently in clinical use and others are under development. Angiotensin-converting (ACE) inhibitors, angiotensin II-receptor blockers (ARB), some β blockers, and calcium channel blockers, all modulate EC function. However, not all have the same clinical effects; for example, ACEI convincingly provide protection from future vascular events, whereas the case for ARB is less clear despite targeting a largely similar pathway.

Compounds that act directly on guanylate cyclase (sGC), a key enzyme of the NO signaling pathway, have also been investigated in preclinical studies. The therapeutic potential of sGC activators (cinaciguat or ataciguat) and sGC stimulators (riociguat) has been explored in animal models and clinical trials (67). In a recent animal study, it was demonstrated that a sGC activator offered renal protection against the progression of nephropathy induced by type 2 diabetes in obese rats (68). However, it remains unclear whether sGC activators can modulate the risk of vascular occlusion in man, and this remains an area for further research.

While all these agents can improve endothelial dysfunction by lowering blood pressure, it is clear that additional mechanisms operate to explain the difference in vascular outcome (69, 70). It has been reported that the thiazolinedione family of hypoglycemic agents, improve endothelial dysfunction in patients with type 2 diabetes by modulating IR (71, 72). Although pioglitazone treatment showed protection from cardiovascular events in diabetes (73), treatment with rosiglitazone therapy was associated with an increase in vascular events (74). This shows that two agents within the same class can have a different clinical outcome, highlighting the difficulties in treating vascular disease in diabetes.

Experimental agents to improve EC function include eNOS-transcription enhancer and agents that inhibit phosphodiesterase-5 (PDE-5) and sphingosine-1-phosphate (75, 76). It has been shown that the eNOS-transcription enhancer AVE3085 restored endothelial function in a hypertensive rat model suggesting that drugs regulating eNOS may be considered as therapeutic targets (77). Furthermore, PDE-5 inhibitors upregulate eNOS expression and, therefore, NO production. It has been reported that these inhibitors can improve endothelial function and control platelet activation in patients with coronary artery disease and have also been shown to improve vascular relaxation in diabetic rats (75, 78). Another target that gained an interest is sphingosine-1-phosphate, which affects the function of cells involved in the atherosclerotic process, including monocyte attachment and proliferation of smooth muscle cells (79).

Aging and diabetes both cause vascular dysfunction and the existence of both conditions has an additive effect in vascular damage, leading to increased vascular inflammation and cardiovascular risk (80). So far, however, there has been little research on the mechanisms of vascular inflammation due to both aging and diabetes, which would be particularly helpful to address vascular disease in older patients with diabetes.

It has recently been demonstrated that following a coronary ischemic event, a single dose of reconstituted HDL increased cardiac glucose uptake and reduced infarct size in both metabolically normal and insulin-resistant mice (81). While these data are interesting, further studies are needed to clarify the role of this approach in modulating vascular risk in man.

Finally, several studies have investigated the role of miRNAs in endothelial dysfunction in diabetes. It has been suggested that these noncoding, single-stranded RNA molecules which are implicated in key processes such as IR and β-cell function, may contribute to the development of prognostic tools or therapeutic targets of CVD in diabetes (82). For example, miR-155 has been implicated with atherosclerosis *via* modulation of actin cytoskeleton organization in ECs, and it was reported that inhibition of miR-155 can reduce atherosclerotic plaques (83). Platelet miR-223 was reduced in patients with diabetes and in mice which had an effect on platelet function. Platelets from miR-223 knockout mice had increased aggregation and potential for thrombus formation compared to platelets from wild-type mice (84, 85).

**Figure 4:**
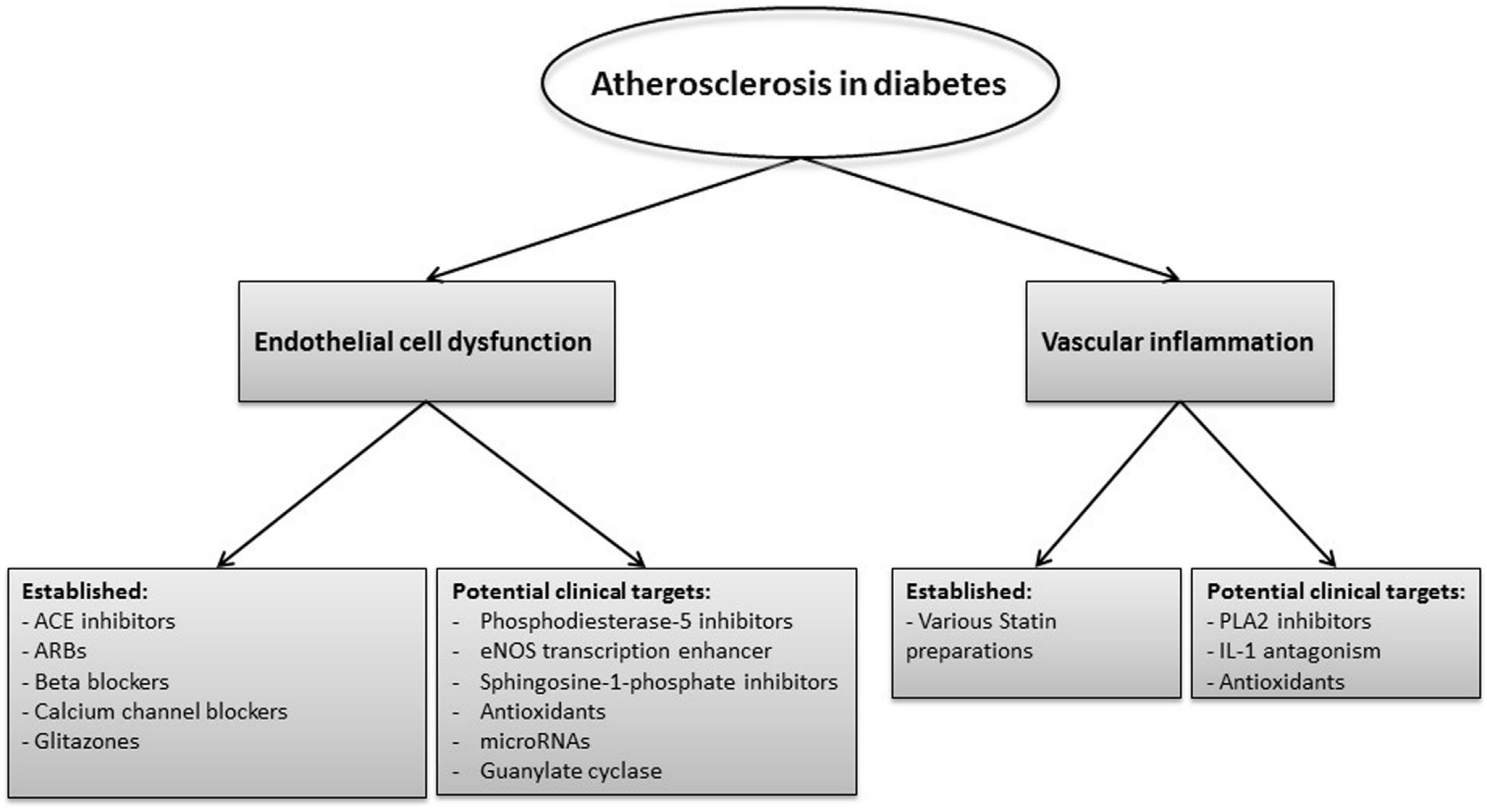
Inflammatory pathways that are currently used or may represent therapeutic targets to reduce vascular risk in diabetes. Current therapies and others in development focus on improving the endothelial cell function and reducing vascular inflammation. Established therapies to target endothelial dysfunction are angiotensin converting (ACE) inhibitors, angiotensin II-receptor blockers (ARBs), beta blockers, calcium channel blockers, and glitazones, whereas agents such as phosphodiesterase-5 inhibitors, endothelial nitric oxide synthase (eNOS) transcription enhancer, and microRNAs represent potential future therapies. Statins are widely used to reduce vascular risk and in addition to their cholesterol lowering effect they reduce vascular inflammation. Other potential targets to reduce vascular inflammation include of phospholipase A2 (PLA2) inhibitors as well as interleukin (IL)-1 antagonists and antioxidants.

The various inflammatory pathways that may represent therapeutic targets to reduce vascular risk in diabetes are summarized in Figure 4.

### Targeting Thrombosis

The thrombotic potential can be modulated both indirectly and directly. The former includes measures to reduce IR, optimize glycemia, and improve endothelial dysfunction. The latter concentrates on antiplatelet therapy as well as agents that target coagulation proteins.

#### Indirect Measures to Improve the Thrombotic Environment

Diabetes-specific measures to improve the thrombotic environment concentrate on ameliorating IR and improving glycemia. This can be achieved by simple measures, such as increased exercise/adhering to a healthy diet through to sophisticated treatment regimen of intensive glycemic control. Improving glycemia reduces platelet activation, modulates fibrin clot structure, and improves efficiency of the fibrinolytic system thus ameliorating the prothrombotic environment in diabetes. However, the short-medium term clinical effects of improving glycemia on vascular events are debatable with studies showing a benefit, no effect and even harm (86–88). The inconsistent results of glycemic studies may be related to precipitation of hypoglycemia with tight glycemic control, which can be prothrombotic. We have shown that an episode of hypoglycemia creates a prothrombotic and hypofibrinolytic environment for at least 1 week (89), explaining the association between hypoglycemia, cardiovascular events, and mortality (90, 91). Therefore, hypoglycemic agents with lower risk of hypoglycemia should be considered in higher vascular risk patients with diabetes. In support of this concept, three relatively recent trials with hypoglycemic agents that are associated with low risk of hypoglycemia have shown improved vascular outcome (92–94). It should be acknowledged, however, that the beneficial effects of these newer hypoglycemia agents are not solely related to avoidance of hypoglycemia and the favorable clinical outcome is likely to be due to modulation of a number of cardiovascular risk factors.

In addition to glycemia, there has been an interest in the antithrombotic properties of omega-3 polyunsaturated fatty acids (n-3 PUFAs). However, a recent study demonstrated that treatment with n-3 PUFAs failed to modulate fibrin clot properties, platelet activation, or inflammation in patients with atherosclerosis and type 2 diabetes (95).

#### Direct Measures to Improve the Thrombotic Environment

Antiplatelet agents remain a cornerstone in the management of the thrombotic environment in diabetes. However, the best treatment regimen at various stages of the vascular pathology is yet to be determined; this can be divided into primary prevention, secondary prevention immediately following an event and long-term secondary prevention. This topic is reviewed extensively elsewhere (26, 60, 96, 97) and we will only provide here a clinically orientated practical summary.

## Antiplatelet Agents

### Primary Prevention

Aspirin irreversibly inhibits cyclo-oxygenase (COX) enzymes, at lower doses exhibiting relative selectivity for COX1, which is responsible for the synthesis of thromboxane A_2_ (TXA_2_), a prothrombotic and vasoconstrictive eicosanoid. At higher doses, aspirin can also inhibit COX2, leading to the unwanted reduction in production of prostacyclin (PGI_2_) an antithrombotic and vasodilatory agent (98). In addition to platelet inhibition, aspirin appears to affect the fibrin network structure and fibrinolysis, which may also contribute to its clinical antithrombotic effects (99).

Until relatively recently, aspirin was used for primary prevention in patients with diabetes, although there was no clear evidence supporting such an approach. A number of studies investigating aspirin in primary vascular protection in diabetes failed to show a benefit, including PPP trial, JPAD, and POPADAD studies, and therefore, current guidelines do not advocate the use of this agent for primary prevention (100–103). It should be acknowledged that none of these studies was adequately powered to give a definitive answer and results from an ongoing large study are currently awaited that will clarify the use of aspirin for primary prevention in diabetes (ASCEND^2^). The guidelines adopted a pragmatic approach, however, by recommending aspirin use for primary prevention in those at higher risk without clearly defining this group.

One of the issues that remain unresolved is the optimal dosing regimen of aspirin in diabetes. Given the increased platelet turnover in diabetes and the relatively short half-life of aspirin, this agent may fail to provide 24 h coverage (97, 104). Therefore, studies have investigated twice daily administration of aspirin, which appears to improve the platelet inhibitory effect, although the clinical value of this approach remains unclear (34–36). This has been alluded to in joint ESC/EASD guidelines but no management recommendations were made due to lack of evidence (103).

### Secondary Cardiovascular Prevention

Aspirin is used in combination with other antiplatelet agents that target the P2Y12 pathway, following acute coronary syndrome (ACS) in diabetes patients and individuals with no diabetes. Of the three agents that target the P2Y12 pathway, both prasugrel and ticagrelor showed superiority to clopidogrel in improving vascular outcome in patients with diabetes (31, 105, 106). A plausible explanation is related to the need to metabolize clopidogrel from the inactive to the active form, which is simpler process with prasugrel and not required at all with ticagrelor.

Dual antiplatelet therapy is usually continued for a period of 1 year after an ACS to be followed by aspirin only therapy that is continued indefinitely. However, recent evidence indicates that longer-term DAPT therapy with aspirin and ticagrelor offers a survival advantage in diabetes patients, unlike those without diabetes (107). The safety and cost effectiveness of this approach requires further analysis and/or studies before routinely recommending long-term DAPT in patients with diabetes (26, 31, 105, 106).

An inherent difficulty in optimizing antiplatelet therapy in diabetes is related to the heterogeneity of this condition and the variable vascular risk according to duration of diabetes, treatment modalities, and presence of microvascular complications. In order to optimize antiplatelet therapy in diabetes, there are a number of questions that remain unanswered, including: (i) Is an alternative dosing of aspirin required in diabetes? (ii) How long to continue treatment with DAT following ACS? (iii) Are different antiplatelet regimen required in different groups of diabetes patients, particularly for primary prevention? (iv) Are additional antiplatelet agents that target other pathways required? Addressing these questions will require large clinical outcome studies which are both time consuming and expensive. Therefore, using surrogate markers, such as platelet activation tests, may provide provisional data justifying clinical outcome studies. In other words, an integrated bench to bedside approach is perhaps the most cost effective way to investigate newer antithrombotic agents or analyze alternative application of existing agents.

## Agents Targeting the Coagulation Pathway

### Established Therapies

Proteins of the contact system have been investigated and considered as therapeutic targets due to their important role in the initiation of thrombosis. TF is expressed by exposed vascular smooth muscle cells at the atherosclerotic plaque rupture sites where it triggers the activation of FVII and the initiation of the coagulation cascade. Activation of FIX and FX by the TF/FVIIa complex then leads to the formation of FXa/FVa complex which converts prothrombin into thrombin (108).

Heparin is an indirect inhibitor of FX and prothrombin and is used in diabetes and non-diabetes subjects with ACS. It is mainly used in unfractionated form or as the low-molecular weight form of heparin, named enoxaparin, which has been suggested to be more effective than the unfractionated form of heparin (109). Another indirect inhibitor of FX which was reported to be as effective as enoxaparin or unfractionated heparin is fondaparinux (110). However, unlike heparin, fondaparinux fails to inhibit thrombin.

The recent development of direct oral anticoagulants has further helped by offering a more targeted inhibition of the protein arm of coagulation. Bivalirudin is a direct inhibitor of thrombin which has been reported to be superior to heparin as a therapy for diabetes patients with ACS due to the significant reduction in bleeding complications in some studies (111, 112). The COMPASS study, involving over 27,000 patients with stable coronary artery disease, has recently shown that combination of rivaroxaban (FX inhibitor) with aspirin is superior to aspirin alone in preventing vascular events, an observation that applied to both patients with and without diabetes (113). However, there was a 70% increase in bleeding risk in patients receiving combination therapy, which is a concern and will have implications for the widespread use of such a therapy.

### Experimental Therapies

FXII and FXI are involved upstream in the coagulation system and have been considered as potential therapeutic targets for thrombosis prevention. Activation of FXII triggers the initiation of the contact system and FXIIa converts prekallikrein to kallikrein. Kallikrein promotes the generation of additional FXIIa and the activation of FXI. Finally, FXIa activates FIX and promotes FX activation and generation of thrombin. The antithrombotic effects of targeting FXII and prekallikrein have been demonstrated *in vivo* using FeCl3- induced and stenosis-induced models of venous thrombosis (114). Different approaches, such as the use of antibodies, small molecules and aptamers have been developed to target factor XII and FXI but the clinical efficacy and safety of such drugs in man are yet to be determined (115).

A crucial element of a successful antithrombotic therapy is maintaining a clinically acceptable risk/benefit ratio. This requires close understanding of the mechanistic pathways operating to increase thrombosis risk in diabetes. For example, it has been shown that both PI and complement C3 incorporation into diabetes clots is enhanced, which further compromises fibrinolysis (detailed above). Developing therapies that reduce incorporation of these proteins into diabetes clots will facilitate fibrinolysis, thus limiting thrombus formation while keeping bleeding risk to a minimum. Therefore, rather than full inhibition of one pathway, which compromises normal physiology and increases bleeding risk, what is perhaps required is partial inhibition of multiple pathways with a focus on those that show a diabetes-specific abnormality.

**Figure 5:**
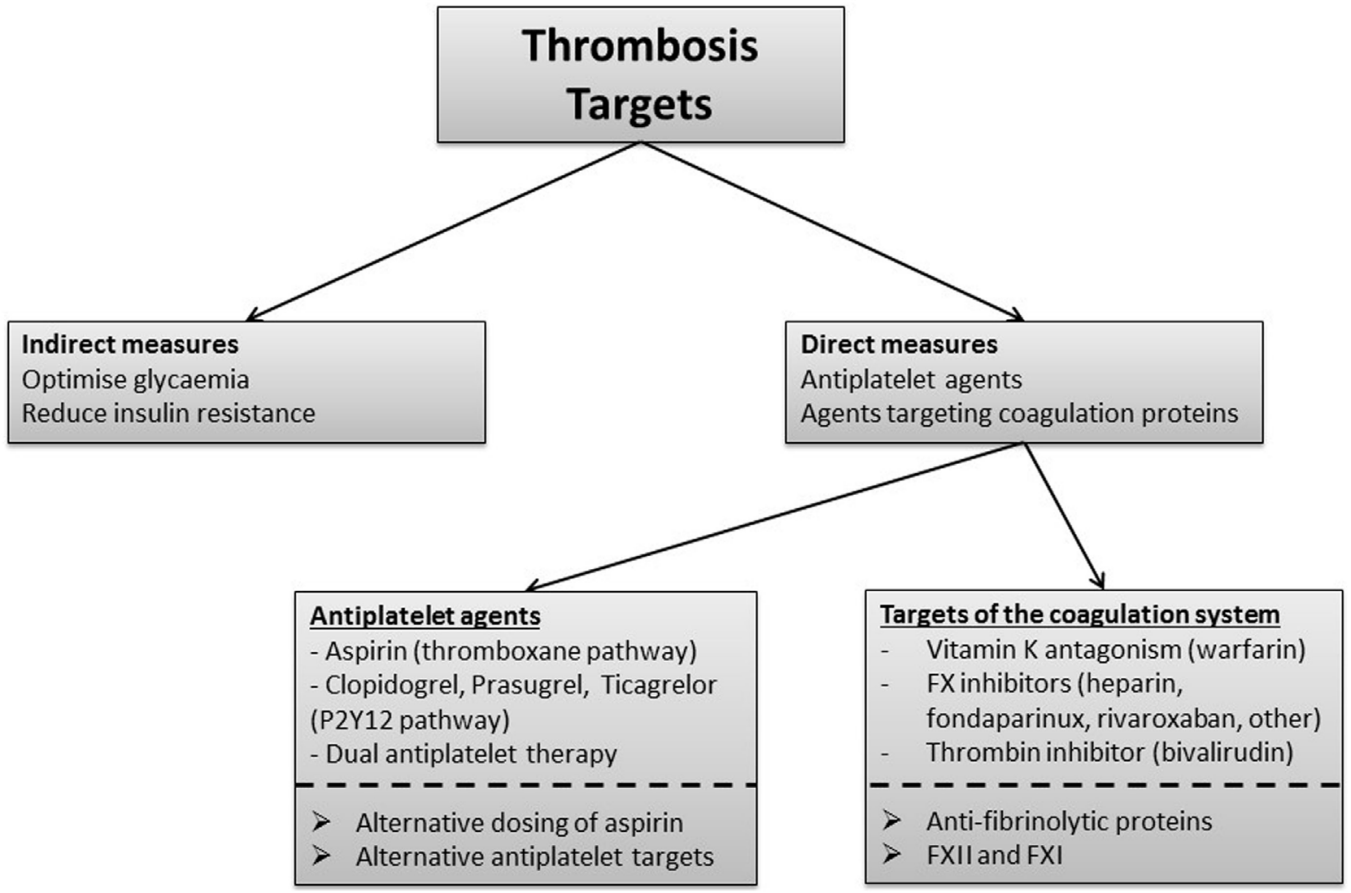
Thrombotic pathways that represent current and potential therapeutic targets in diabetes. Indirect measures to improve the thrombotic environment in diabetes focus on optimizing glycemic control by lowering glucose levels while avoiding hypoglycemia and also by reducing insulin resistance. Antiplatelets and agents that target the coagulation system comprise the main direct measures to manage the thrombotic environment in diabetes. Aspirin targets the thromboxane pathway and is also used in combination with clopidogrel, prasugrel, or ticagrelor which target the P2Y12 pathway. Alternative antiplatelet targets are under consideration. Vitamin K antagonism reduces thrombosis risk but is associated with high risk of bleeding when combined with antiplatelet therapy, and therefore, not routinely used for atherothrombotic disease. Inhibitors of coagulation proteins FX and thrombin, such as heparin and direct oral anticoagulants, represent agents with promise in diabetes while targeting factor (F)XII and FXI requires careful future evaluation. Alternative dosing of aspirin and reduction of incorporation of antifibrinolytic proteins into the clot may represent new approaches to improve clinical outcome in patients with diabetes.

Potential treatments and targets to further reduce atherothrombosis risk in diabetes are summarized in Figure 5.

## Conclusion and the Way Forward

Research conducted for the prevention and treatment of atherothrombotic disease has seen major advances over the past few decades, which translated clinically into significant improvement in vascular mortality and morbidity. We now have a number of agents that limit the atherosclerotic process and also control the risk of thrombosis. Despite these advances, however, the risk of vascular disease in patients with diabetes remains unacceptably high with relatively poor prognosis following an ischemic event. Therefore, future work is required to develop more effective therapeutic agents to halt the accelerated atherosclerotic process, typically associated with diabetes. Moreover, work is needed to develop antithrombotic strategies that target diabetes-specific pathways, thereby maximizing benefit and limiting the risk of bleeding. These include alternative use of existing agents and development of novel agents. An example of the former is investigating the efficacy of twice daily aspirin regimen in diabetes, whereas the latter includes development of agents that target the hypofibrinolytic environment, a key abnormality in individuals with deranged glucose metabolism (44).

Finally, we should accept that diabetes is not a single clinical entity but a continuum of different stages of the condition with each having a different vascular risk. Therefore, in order to maximize benefit, future therapies aiming to reduce atherothrombotic risk in diabetes may require stratifying patients into different categories with each group having a “stage-specific” vascular management strategy. Admittedly, this will add to the complexity of clinical therapy, particularly as vascular risk will vary over time in each patient, but this “individualized care” approach is perhaps key to improve long-term outcome in this high-risk population.

## Author Contributions

NP designed the review, undertook the literature search, and wrote the manuscript and RA designed the review and critically reviewed the manuscript. Both authors agreed to the final manuscript.

## Conflict of Interest Statement

The authors declare that the research was conducted in the absence of any commercial or financial relationships that could be construed as a potential conflict of interest.

## Footnotes

^1^http://www.who.int.

^2^https://clinicaltrials.gov/ct2/show/NCT00135226.
